# Sumoylation of Hypoxia-Inducible Factor-1α Ameliorates Failure of Brain Stem Cardiovascular Regulation in Experimental Brain Death

**DOI:** 10.1371/journal.pone.0017375

**Published:** 2011-03-03

**Authors:** Julie Y. H. Chan, Ching-Yi Tsai, Carol H. Y. Wu, Faith C. H. Li, Kuang-Yu Dai, Enya Y. H. Sun, Samuel H. H. Chan, Alice Y. W. Chang

**Affiliations:** 1 Department of Medical Education and Research, Kaohsiung Veterans General Hospital, Kaohsiung, Taiwan, Republic of China; 2 Center for Translational Research in Biomedical Sciences, Chang Gung Memorial Hospital-Kaohsiung Medical Center, Kaohsiung, Taiwan, Republic of China; University of Colorado Denver, United States of America

## Abstract

**Background:**

One aspect of brain death is cardiovascular deregulation because asystole invariably occurs shortly after its diagnosis. A suitable neural substrate for mechanistic delineation of this aspect of brain death resides in the rostral ventrolateral medulla (RVLM). RVLM is the origin of a life-and-death signal that our laboratory detected from blood pressure of comatose patients that disappears before brain death ensues. At the same time, transcriptional upregulation of heme oxygenase-1 in RVLM by hypoxia-inducible factor-1α (HIF-1α) plays a pro-life role in experimental brain death, and HIF-1α is subject to sumoylation activated by transient cerebral ischemia. It follows that sumoylation of HIF-1α in RVLM in response to hypoxia may play a modulatory role on brain stem cardiovascular regulation during experimental brain death.

**Methodology/Principal Findings:**

A clinically relevant animal model that employed mevinphos as the experimental insult in Sprague-Dawley rat was used. Biochemical changes in RVLM during distinct phenotypes in systemic arterial pressure spectrum that reflect maintained or defunct brain stem cardiovascular regulation were studied. Western blot analysis, EMSA, ELISA, confocal microscopy and immunoprecipitation demonstrated that drastic tissue hypoxia, elevated levels of proteins conjugated by small ubiquitin-related modifier-1 (SUMO-1), Ubc9 (the only known conjugating enzyme for the sumoylation pathway) or HIF-1α, augmented sumoylation of HIF-1α, nucleus-bound translocation and enhanced transcriptional activity of HIF-1α in RVLM neurons took place preferentially during the pro-life phase of experimental brain death. Furthermore, loss-of-function manipulations by immunoneutralization of SUMO-1, Ubc9 or HIF-1α in RVLM blunted the upregulated nitric oxide synthase I/protein kinase G signaling cascade, which sustains the brain stem cardiovascular regulatory machinery during the pro-life phase.

**Conclusions/Significance:**

We conclude that sumoylation of HIF-1α in RVLM ameliorates brain stem cardiovascular regulatory failure during experimental brain death via upregulation of nitric oxide synthase I/protein kinase G signaling. This information should offer new therapeutic initiatives against this fatal eventuality.

## Introduction

As much as brain death is currently the legal definition of death in the United States [1], European Union [2] or Taiwan [3] and is of paramount clinical importance, its cellular and molecular underpinning remains relatively unsettled. Despite the complexity of brain death, two clinical observations suggest that mechanistic delineation of permanent impairment of the brain stem cardiovascular regulatory machinery associated with this fatal phenomenon may shed light on its underlying mechanisms. First, asystole invariably takes place within hours or days after the diagnosis of brain death [4]. Second, a unique clinical phenotype exists in the low-frequency (LF) component (0.04–0.15 Hz in human) of the systemic arterial pressure (SAP) spectrum, which reflects the status of brain stem cardiovascular regulation. The power density of the LF component undergoes a dramatic reduction or loss before brain death ensues in comatose patients [5]–[7]. That this life-and-death signal originates from the rostral ventrolateral medulla (RVLM) [8], which is known classically for its role in tonic maintenance of vasomotor tone and SAP [9], allows this brain stem site to be a suitable neural substrate for mechanistic assessment of the brain stem cardiovascular regulatory functions associated with brain death [10].

In an animal model that employs mevinphos (3-[dimethoxyphosphinyl-oxyl]-2-butenoic acid methyl ester; Mev) as the experimental insult [10]–[12], our laboratory found previously that on systemic administration, this organophosphate pesticide acts directly on RVLM to elicit an augmentation followed by a reduction of the life-and-death signal [11]–[16]. Since this temporal pattern resembles that exhibited during the progression towards brain death by patients died of organophosphate poisoning [6], they are designated respectively the pro-life and pro-death phase in this model of brain death [10]. We further demonstrated that nitric oxide (NO) generated by NO synthase I (NOS I) in RVLM, followed by activation of the soluble guanylyl cyclase/cGMP/ protein kinase G (PKG) cascade, is responsible for the pro-life phase; peroxynitrite formed by a reaction between NOS II-derived NO and superoxide anion underlies the pro-death phase [13], [14].

That Mev elicits hypoxia in RVLM [15] and that both NOS I [17], [18] and NOS II [19], [20] are hypoxia responsive gene products offer an important clue in our continual search for the cellular and molecular mechanisms that underlie brain death. Because of the reduction in the amount of oxygen that reaches brain tissues, hypoxia in brain tissue may trigger a series of adaptive responses in neurons. In this regard, the transcription factor hypoxia-inducible factor 1 (HIF-1) operates as a master regulator of cellular responses to hypoxia via activation of a multitude of oxygen-sensitive gene products [21]–[24]. We demonstrated recently [25] that one of the hypoxia responsive gene products upregulated transcriptionally by HIF-1α [26] in RVLM is heme oxygenase-1, which plays a pro-life role in experimental brain death by sustaining central cardiovascular regulatory function via activation of heat shock protein 70 [27]. A new system of post-translational protein modification that is also activated by transient cerebral ischemia [28], [29] or hypoxia [30], in addition to phosphorylation and ubiquitin conjugation, is massive protein conjugation by the small ubiquitin-like modifier (SUMO). Of note is that HIF-1α is among the many transcription factors that are sumoylated [31]. Acute hypoxia induces expression of SUMO-1 [32], although the effects of sumoylation of HIF-1α on its stability and transcriptional activity are controversial [33]–[36]. It follows that sumoylation of HIF-1α in RVLM in response to hypoxia may play a modulatory role on brain stem cardiovascular regulation during experimental brain death.

The present study assessed the hypothesis that sumoylation of HIF-1α in RVLM participates in brain stem cardiovascular regulation during brain death by interacting with NOS I/PKG or NOS II/peroxynitrite signaling pathway. We demonstrated that sumoylation of HIF-1α in RVLM plays a preferential pro-life role by ameliorating cardiovascular regulatory failure during brain death via selective upregulation of NOS I/PKG signaling cascade.

## Results

### Biphasic cardiovascular responses in experimental brain death

**Figure 1:**
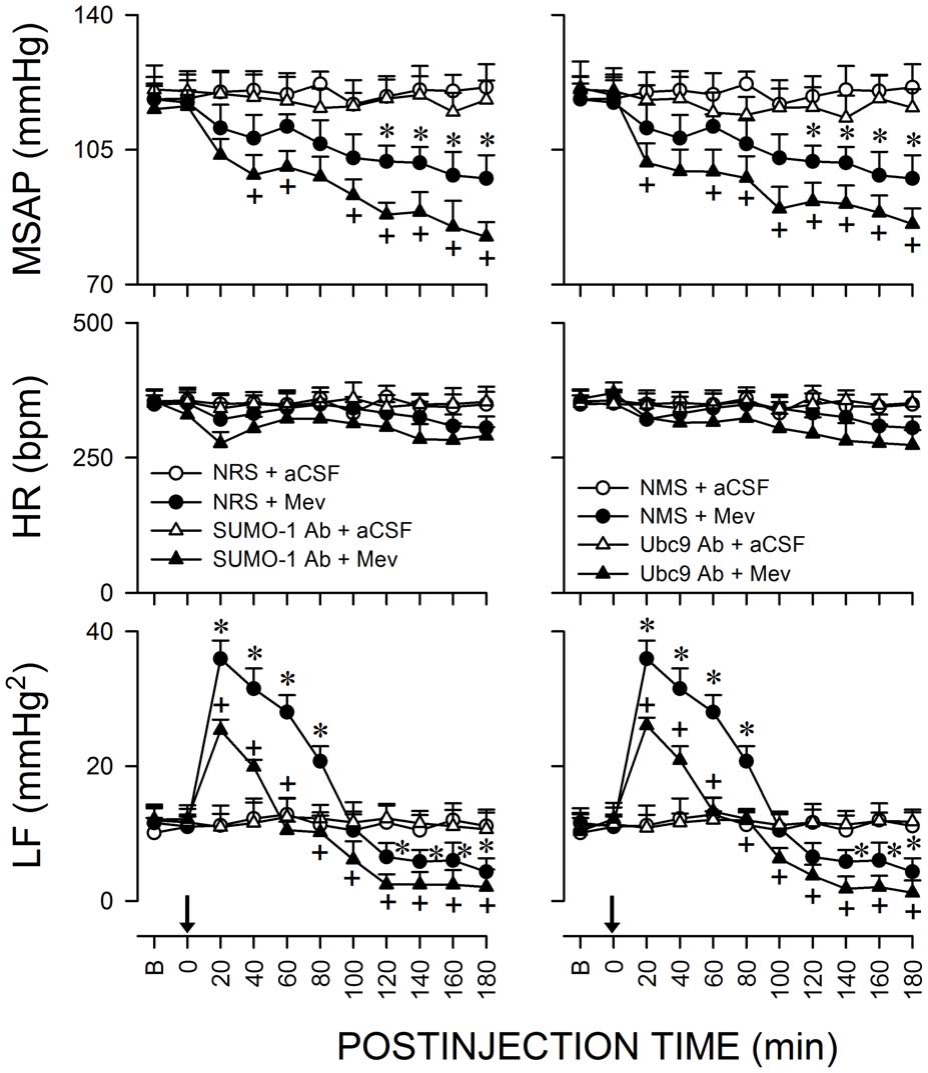
Sumoylation of proteins in RVLM ameliorates failure of central cardiovascular regulation in Mev intoxication model of brain death. Temporal changes in mean systemic arterial pressure (MSAP), heart rate (HR) or power density of the low-frequency (LF) component of SAP signals in rats that received pretreatment by microinjection bilaterally into RVLM of an anti-SUMO-1 or anti-Ubc9 antiserum (1∶20), normal rabbit serum (NRS,1∶20; vehicle for anti-SUMO antiserum) or normal mouse serum (NMS, 1∶20; vehicle for anti-Ubc9 antiserum), 30 min before local application (at arrow) of artificial cerebrospinal fluid (aCSF) or mevinphos (Mev; 10 nmol) to the bilateral RVLM. Values are mean ± SEM, n = 5–7 animals per experimental group. *P<0.05 versus NRS+aCSF or NMS+aCSF group, and ^+^ *P*<0.05 versus NRS+Mev or NMS+Mev group at corresponding time-points in the post hoc Scheffé multiple-range test. B, baseline.

Figure 1 shows that microinjection bilaterally of Mev (10 nmol) into RVLM in animals pretreated with vehicle elicited a progressive hypotension that became significant 100 min after application, accompanied by indiscernible alterations in heart rate (HR). Concurrent changes in power density of the LF component of SAP signals revealed two distinct phases of Mev-induced cardiovascular responses. The pro-life Phase I entailed a significantly augmented LF power that endured 80–100 min. The pro-death Phase II, which lasted the remainder of our 180-min observation period, exhibited further and significant reduction in the power density of this spectral component to below baseline to reflect failure of brain stem cardiovascular regulatory functions that precedes brain death [10].

### Sumoylation of proteins in RVLM ameliorates failure of brain stem cardiovascular regulation associated with experimental brain death

The most fundamental premise for the present study is that sumoylation of proteins in RVLM is causally involved in brain stem cardiovascular regulation during brain death. Our first series of experiments employed loss-of-function by immunoneutralization to establish this premise. Figure 1 shows that compared to normal rabbit or mouse serum, pretreatment with microinjection bilaterally into RVLM of an antiserum (1∶20) against SUMO-1 or Ubc9, the only known conjugating enzyme for the sumoylation pathway [37], [38] 30 min before local application of Mev (10 nmol), significantly and selectively enhanced the progressive hypotension and reduced the increase in power density of LF component of SAP signals during Phase I Mev intoxication, without affecting HR. In addition, the hypotension and reduced LF power already exhibited during Phase II were significantly potentiated.

### Preferential augmentation of protein sumoylation in RVLM during the pro-life phase

**Figure 2:**
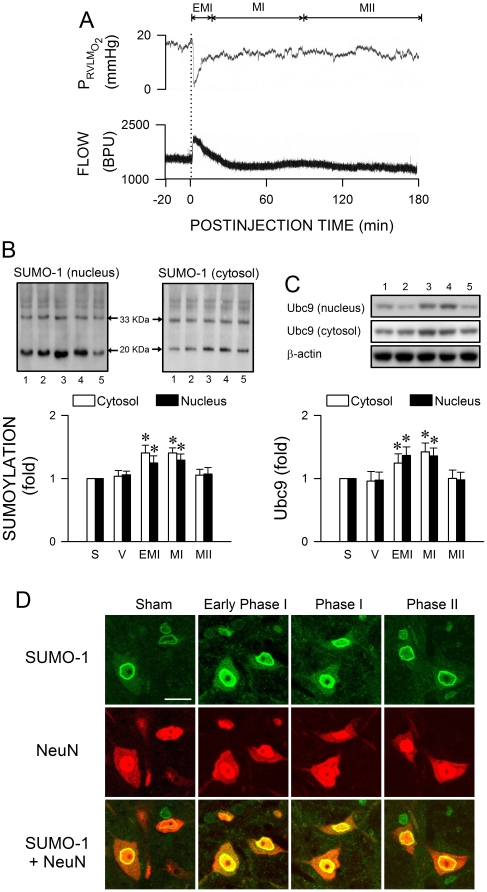
Severe hypoxia and preferential augmentation of protein sumoylation in RVLM during the pro-life phase. (*A*) Representative continuous tracings showing temporal changes of tissue oxygen concentration (upper panel) or microvascular perfusion (lower panel) in RVLM of rats that received intravenous administration of Mev (640 µg kg^−1^, at dashed line) during early Phase I (EMI) and Phases I and II (MI and MII) Mev intoxication. BPU: blood perfusion unit. (*B* and *C*) Illustrative gels or summary of fold changes against sham (S) controls of nuclear or cytosolic proteins that were conjugated to SUMO-1 (*B*) or Ubc9 protein expression (*C*) detected in ventrolateral medulla during Phases EMI, MI and MII Mev intoxication or in aCSF (V) controls. Since comparable results were obtained from all corresponding time intervals after animals received microinjection of aCSF, only one set of data is presented in this and subsequent figures for clarity. Values are mean ± SEM of triplicate analyses on samples pooled from 4–6 animals in each group. *P<0.05 versus aCSF group in the post hoc Scheffé multiple-range analysis. Note numbers on bottom of the gels correspond to columns in the data summary. (*D*) Representative laser scanning confocal microscopic images showing cells in RVLM that were immunoreactive to SUMO-1 (green fluorescence) and additionally stained positively for a neuronal marker, neuron-specific nuclear protein (NeuN; red fluorescence) in sham controls or during Phases EMI, MI and MII Mev intoxication. These results are typical of 4 animals from each experimental group. Scale bar, 20 µm.

We next evaluated the logical possibility that the augmentation of sumoylated proteins in RVLM underpins its ameliorating effect on Mev-elicited central cardiovascular regulatory dysfunction, noting that acute hypoxia induces expression of SUMO-1 [32]. In this particular set of experiments, Mev was given intravenously because the dimension of this neural substrate does not allow for simultaneous placement of the glass micropipette used for microinjection and the probe used for sensing tissue oxygen and blood flow. Our laboratory demonstrated previously [16] that systemic administration of Mev induces cardiovascular responses by acting on RVLM. Given at an i.v. dose of 640 µg kg^−1^, the elicited changes in SAP, HR and LF power were compatible both in magnitude and time-course with those induced by microinjection of Mev (10 nmol) directly to RVLM. Figure 2A shows that the tissue oxygen tension in RVLM underwent, after a short latency, a drastic drop by 12±5 mmHg (n = 7) that lasted 8–10 min after i.v. administration of Mev. This was followed by a sustained hypoxia at 4±2 mmHg (n = 7) below baseline over the remainder of our 180-min observation period. Intriguingly, the local blood flow responded with an increase that mirrored the abrupt hypoxia, followed by a sustained reduction of approximately 550±60 blood perfusion units (BPU) (n = 7).

Western blot analysis (Figure 2B) revealed a significant augmentation of proteins that were conjugated to SUMO-1 in both cytosolic and nuclear fractions of extracts from ventrolateral medulla during early Phase I when the drastic drop in tissue oxygen level in RVLM took place. This increase sustained throughout Phase I, and returned to baseline during Phase II Mev intoxication. Ubc9 exhibited similar temporal expression pattern (Figure 2B). On the other hand, the level of proteins conjugated by SUMO-1 or expression of Ubc9 in ventrolateral medulla of animals that received artificial cerebrospinal fluid (aCSF) was comparable to sham controls (Figure 2B and 2C). Double immunofluorescence staining coupled with laser scanning confocal microscopy revealed that the results on SUMO-1 demonstrated in our biochemical analyses on protein extracts from ventrolateral medulla indeed took place at the neuronal level. Against a clearly defined nucleus and nucleolus in cells stained positively with the neuronal marker, neuron-specific nuclear protein (NeuN), there was an increased presence of SUMO-1-immunoreactivity in the cytoplasm and nucleus of RVLM neurons during early and late Phase I when compared with sham control or aCSF control (data not shown), which subsided during Phase II (Figure 2D).

### Activation of HIF-1α in RVLM also ameliorates failure of brain stem cardiovascular regulation associated with experimental brain death

**Figure 3:**
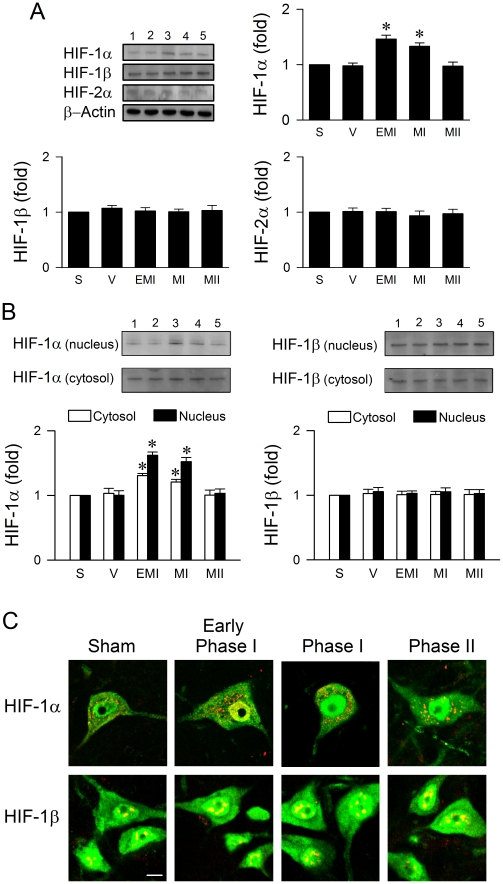
Preferential nucleus-bound translocation of HIF-1α in RVLM during the pro-life phase. (*A* and *B*) Illustrative gels or summary of fold changes against sham (S) controls in ratio of total protein of HIF-1α, HIF-1β or HIF-2α relative to β-actin protein (*A*), or nuclear or cytosolic content of HIF-1α or HIF-1β protein (*B*) detected in ventrolateral medulla during Phases EMI, MI and MII Mev intoxication or in aCSF (V) controls. Values are mean ± SEM of triplicate analyses on samples pooled from 4–6 animals in each group. *P<0.05 versus aCSF group in the post hoc Scheffé multiple-range analysis. Note numbers on top of the gels correspond to columns in the data summary. (*C*) Representative laser scanning confocal microscopic images showing cells in RVLM that were immunoreactive to NeuN (green fluorescence) and additionally stained positively for HIF-1α or HIF-1β subunit (yellow fluorescence or colocalization of red and green fluorescence) in sham controls or during Phases EMI, MI and MII Mev intoxication. These results are typical of 4 animals from each experimental group. Scale bar, 8 µm.

Our third series of experiments investigated whether HIF-1α, which is potently activated by hypoxia [22]–[24], also participates in brain stem cardiovascular regulation during experimental brain death. Western blot analysis (Figure 3A) on total proteins extracted from ventrolateral medulla revealed that whereas HIF-1β or HIF-2α expression remained constant, HIF-1α level was significantly elevated during early Phase I when drastic drop in tissue oxygen level in RVLM took place. This augmented HIF-1α expression sustained throughout Phase I, and returned to baseline during Phase II Mev intoxication. HIF-1α, HIF-1β or HIF-2α expression in ventrolateral medulla of animals that received aCSF was comparable to sham controls (Figure 3A). Detailed immunoblot analysis (Figure 3B) further established a significant elevation of HIF-1α in both the nuclear and cytosolic fractions of proteins extracted from ventrolateral medulla during Phase I Mev intoxication. On the other hand, the primarily nuclear presence of HIF-1β subunit (Figure 3B) or cytosolic presence of HIF-2α (data not shown) remained constant. Again, double immunofluorescence staining coupled with laser scanning confocal microscopy revealed that immunoreactivity of HIF-1α (Figure 3C) was identified primarily in the cytoplasm and HIF-1β in the nucleus of RVLM neurons in sham-control animals. During early and late Phase I, the abundant presence of HIF-1α immunoreactivity in the nucleus of RVLM neurons (Figure 3C) was accompanied by an augmented amount in the cytoplasm, which subsided during Phase II. On the other hand, there was minimal alteration in the preferential presence of HIF-1β immunoreactivity in the nucleus (Figure 3C) of RVLM neurons during both phases of Mev intoxication.

**Figure 4:**
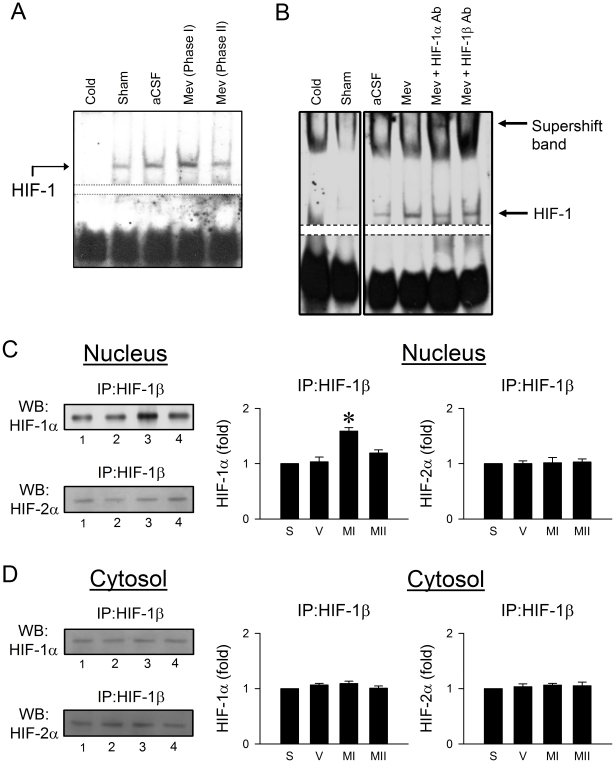
Preferential augmentation of HIF-1α transcription activity in RVLM during the pro-life phase. (*A* and *B*) Illustrative gels depicting HIF-1 DNA binding in nuclear extracts from ventrolateral medulla of sham or aCSF controls, or during Mev intoxication. Results from supershift analysis illustrated in (*B*) were obtained from nuclear extracts from ventrolateral medulla collected during Phase I that was preincubated with antiserum against HIF-1α or HIF-1β. Competitive assay using unlabeled HIF oligonucleotide served as the negative control (Cold). These results are typical of 4 animals from each experimental group. (*C* and *D*) Illustrative gels or summary of fold changes against sham (S) controls of HIF-1α or HIF-2α from proteins immunoprecipitated by anti-HIF-1β antiserum in the nuclear (*C*) or cytosolic (*D*) fraction of samples collected from ventrolateral medulla of sham (S) or aCSF (V) controls or during Phases MI and MII Mev intoxication. Values are mean ± SEM of triplicate analyses on samples pooled from 4–6 animals in each group. *P<0.05 versus aCSF group in the post hoc Scheffé multiple-range test.

**Figure 5:**
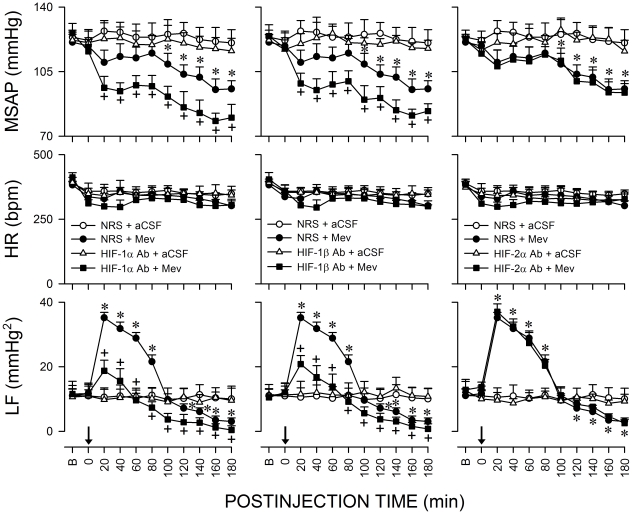
Activation of HIF-1α in RVLM ameliorates failure of central cardiovascular regulation associated with experimental brain death. Temporal changes in MSAP, HR or power density of LF component of SAP signals in rats that received pretreatment by microinjection bilaterally into RVLM of NRS (1∶20), anti-HIF-1α, HIF-1β or HIF-2α antiserum (1∶20), 30 min before local application (at arrow) of aCSF or Mev (10 nmol) to the bilateral RVLM. Values are mean ± SEM, n = 5–7 animals per experimental group. *P<0.05 versus NRS+aCSF group, and ^+^ *P*<0.05 versus NRS+Mev group at corresponding time-points in the post hoc Scheffé multiple-range test. B, baseline.

Nucleus-bound translocation of HIF implies enhanced transcription activity via the formation of heterodimer between HIF-1β and HIF-1α or HIF-2α in the nucleus [39]–[41]. Results from electrophoresis mobility shift assay (EMSA) (Figure 4A and 4B) demonstrated a significant increase in the association of HIF-1 with its consensus DNA oligonucleotide in nuclear extracts from ventrolateral medulla during the pro-life Phase I, which returned during the pro-death Phase II to levels exhibited by sham or aCSF controls. We confirmed that this association was not due to non-specific binding when a competitive assay using unlabeled oligonucleotide probe resulted in discernible disappearance of HIF-1 DNA binding (Figure 4A and 4B). Supershift experiments (Figure 4B) revealed that an antiserum against HIF-1α, but not HIF-1β or HIF-2α (data not shown), retarded the migration of proteins that interacted with the double-stranded HIF-1 oligonucleotide. Immunoblot analysis of proteins immunoprecipitated by an anti-HIF-1β antiserum from the nuclear fraction of ventrolateral medullary samples additionally confirmed a preferential augmentation in the complex formed between HIF-1β and HIF-1α during the pro-life phase of Mev intoxication (Figure 4C). On the other hand, the association between HIF-1β and HIF-2α during both phases was unaffected (Figure 4C). As a control (Figure 4D), minimal association between HIF-1β and HIF-1α or HIF-2α was detected from the cytosolic fraction during both phases. The activation of HIF-1α in RVLM is causally involved in central cardiovascular regulation in our model of brain death (Figure 5). Immunoneutralization of HIF-1α or HIF-1β in RVLM significantly enhanced the progressive hypotension and antagonized the increase in power density of LF component of SAP signals during Phase I Mev intoxication, without affecting HR. Furthermore, the hypotension and reduced LF power already exhibited during Phase II was again significantly augmented. On the other hand, pretreatment with the same dose of anti-HIF-2α antiserum (Figure 5) was ineffective against the phasic cardiovascular responses induced by Mev.

### Preferential sumoylation of HIF-1α in RVLM during the pro-life phase

**Figure 6:**
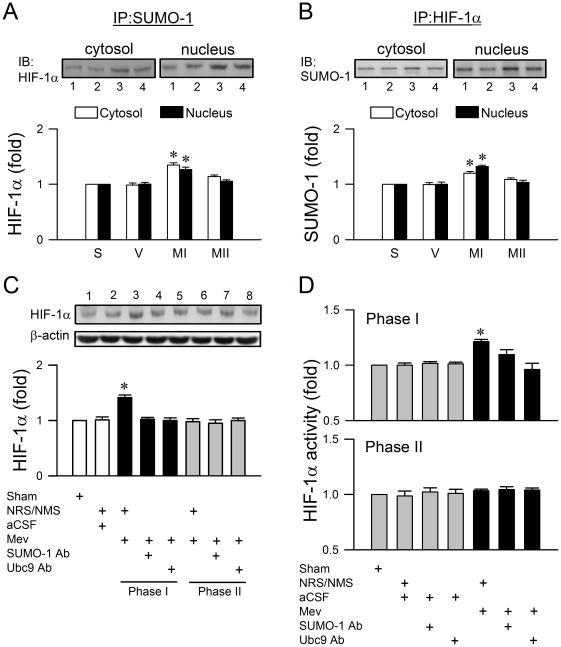
Preferential sumoylation of HIF-1α in RVLM during the pro-life phase. (*A* and *B*) Illustrative gels or summary of fold changes against sham (S) controls of HIF-1α from proteins immunoprecipitated by anti-SUMO-1 antiserum (*A*); or SUMO-1 from proteins immunoprecipitated by anti-HIF-1α antiserum (*B*) in the nuclear or cytosolic fraction of samples collected from ventrolateral medulla of sham (S) or aCSF (V) controls during Phases MI and MII Mev intoxication. (*C* and *D*) Illustrative gels or summary of fold changes against sham (S) controls in ratio of HIF-1α relative to β-actin protein in whole cell lysate (*C*) or transcriptional activity of HIF-1α in nuclear extract measured by an ELISA-based HIF-1 transcription factor assay kit (*D*) that detects the amount of HIF-1 binding to an oligonucleotide containing the hypoxia response element sequence from rats that received pretreatment by microinjection bilaterally into RVLM of an anti-SUMO-1 or anti-Ubc9 antiserum (1∶20), NRS (1∶20) or NMS (1∶20), 30 min before local application of aCSF or Mev (10 nmol) to the bilateral RVLM. Values are mean ± SEM of triplicate analyses on samples pooled from 4–6 animals in each group. *P<0.05 versus aCSF, NRS or NMS group in the post hoc Scheffé multiple-range test. Note numbers below (*A, B*) or on top (*C*) of the gels correspond to columns in the data summary.

As a well-documented target for SUMO-1 [33]–[36], our fourth series of experiments evaluated whether augmentation of sumoylation of HIF-1α is causally related to its enhanced stability or transcriptional activity in RVLM during the pro-life phase. Results from immunoblot analysis of HIF-1α in proteins immunoprecipitated by SUMO-1 showed a preferential increase during Phase I Mev intoxication in the association of SUMO-1 and HIF-1α in both the nuclear and cytosolic fractions from ventrolateral medulla (Figure 6A). Comparable results were obtained from immunoblot analysis of SUMO-1 from proteins immunoprecipitated by HIF-1α (Figure 6B). Furthermore, microinjection bilaterally of an anti-SUMO-1 or anti-Ubc9 antiserum (1∶20) in RVLM significantly blunted the preferentially augmented HIF-1α expression (Figure 6C) and antagonized the heightened transcription activity of HIF-1α in whole cell lysate of ventrolateral medulla during this pro-life phase (Figure 6D).

### Activation of HIF-1 leads to phasic upregulation of NOS I/PKG signaling in RVLM

**Figure 7:**
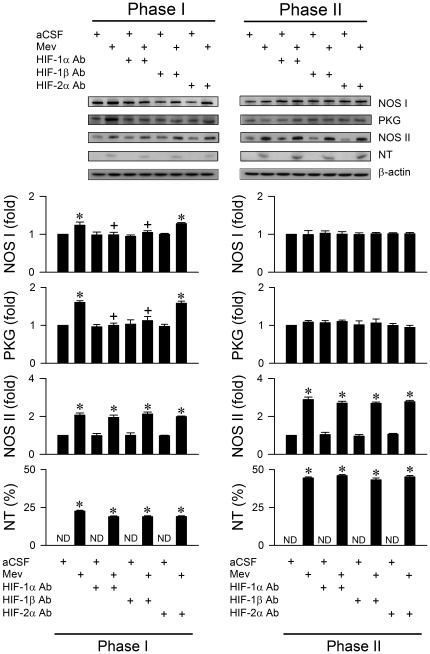
Activation of HIF-1 leads to upregulation of NOS I/PKG signaling in RVLM during the pro-life phase. Illustrative gels or summary of fold changes against aCSF controls in ratio of nitric oxide synthase I (NOS I), protein kinase G (PKG), NOS II or nitrotyrosine (NT; marker for peroxynitrite) relative to β-actin protein detected in ventrolateral medulla of rats that received immunoneutralization of HIF-1α, HIF-1β or HIF-2α subunit in RVLM, 30 min before induction of Mev intoxication. Note that NT is presented as % relative to β-actin because it is below detection limit (ND) in aCSF controls. Values are mean ± SEM of triplicate analyses on samples pooled from 4–6 animals per experimental group. *P<0.05 versus aCSF group and ^+^ *P*<0.05 versus Mev group in the post hoc Scheffé multiple-range test.

We demonstrated previously [11]–[14] that whereas NOS I/PKG signaling in RVLM is responsible for the pro-life phase, NOS II/peroxynitrite signaling underlies the pro-death phase of Mev intoxication. Our final series of experiments assessed whether the upregulation of HIF-1 may subserve its pro-life role via modulation of these two signaling pathways. Immunoneutralization of HIF-1α or HIF-1β (Figure 7) blunted significantly and selectively the Mev-induced Phase I increase in NOS I or PKG protein expression in ventrolateral medulla. None of these pretreatments affected the progressive increase in NOS II or nitrotyrosine (marker for peroxynitrite) during both phases of Mev intoxication. Again, anti-HIF-2α antiserum was ineffective (Figure 7) against the phasic Mev-induced NOS I, PKG, NOS II or nitrotyrosine protein expression in ventrolateral medulla.

## Discussion

HIF-1 activation plays an important role in tissue preservation as a response to regional hypoxia [42]. Of the very limited amount of information available on the functional role of HIF-1 in the brain, HIF-1α activation in cortical neurons produces rescue proteins in response to intermittent hypoxia [43]. HIF-1 is a heterodimer of two basic helix-loop-helix/PAS proteins, HIF-1α and HIF-1β [40]. HIF-1α is rapidly degraded under normoxic conditions by the ubiquitin-proteasome system, and HIF-1β is constitutively expressed in the nucleus and its level is not significantly affected by oxygen levels [41]. Hypoxia stabilizes HIF-1α, and nucleus-bound translocation of the stabilized HIF-1α allows for formation of the HIF-1αβ heterodimer that becomes transcriptionally active [39]. Jewell et al. [44] showed that human epithelial carcinoma cell line HeLaS3 already exhibited nuclear HIF-1α protein induction and HIF-1 DNA binding after the introduction of hypoxia for less than 2 min, reaching maximal levels one hour after anoxic/hypoxic exposure. Based on complementary results from Western blot analysis, EMSA, confocal microscopy and immunoprecipitation, we demonstrated that the repertoire of cellular events that included nucleus-bound translocation of HIF-1α, followed by formation of HIF-1αβ heterodimer in RVLM neurons during the pro-life phase of Mev intoxication exhibited a time-course that paralleled the elicited drastic hypoxia in RVLM. More importantly, loss-of-function manipulations in conjunction with monitors of circulatory events showed that the activated HIF-1α ameliorated brain stem cardiovascular regulatory failure during the progression towards experimental brain death. We further found that HIF-2α plays no part in this process.

A novel contribution of the present study is the identification of a vital role for sumoylation of HIF-1α in RVLM in ameliorating brain stem cardiovascular regulatory dysfunction during brain death. The covalent conjugation of SUMOs to proteins has received much attention since its discovery [45], [46] because the affected proteins are involved in gene expression, chromatin structure, signal transduction or maintenance of the genome [31]. Transient global cerebral ischemia induces massive increases in sumoylation of proteins, including transcription factors [29]. In particular, hypoxia increases SUMO-1 mRNA and protein levels in brain, and the induced SUMO-1 co-expresses with HIF-1α in neurons [32]. However, controversy exists on the cellular sequel of modulation of HIF-1α through sumoylation. It is generally contended that sumoylation of transcription factors blocks their activation [37], [47]. Thus, conjugation of SUMO to HIF-1α decreases its activity [34], and hypoxia-induced sumoylation of HIF-1α leads to its ubiquitination and degradation [36]. On the other hand, Bae et al. [33] reported that upregulation of HIF-1α through SUMO-1 modification at Lys(391)/Lys(477) residues increases its stability and enhances its transcriptional activity, and sumoylation of HIF-1α blocks its degradation [29]. Whereas the present study was not designed to address this controversy, complementary results from our biochemical and physiological experiments strongly indicated that elevation in SUMO-1 and Ubc9 levels that paralleled temporally with hypoxia, followed by an increase in conjugation between SUMO-1 and HIF-1α, took place in RVLM preferentially during the pro-life phase. More importantly, we demonstrated in our model that this enhanced sumoylation of HIF-1α in RVLM ameliorates the dysfunction of brain stem cardiovascular regulation associated with brain death. The return of SUMO-1, Ubc9 or HIF-1α expression in RVLM to control levels during phase II despite a sustained oxygen level at 4±2 mmHg below baseline suggests that stronger hypoxic drive is required to trigger sumoylation of HIF-1α and its transcriptional activities. Although unrelated directly to brain death, the engagement of sumoylation in cardiovascular regulation is reported in a recent study [48]. Sumoylation of phosducin inhibits postganglionic sympathetic neuron activity and prevents stress-induced hypertension.

Gill [49] reviewed that SUMO shares structural homology with ubiquitin, and the machinery that attaches SUMO to substrate proteins and the enzymes that participate in ubiquitination are strikingly similar. As such, SUMO may compete with ubiquitin for the same lysine residues and function essentially as an antiubiquitin [47]. We reason that this modus operandi is at work in RVLM during brain death, noting that degradation of HIF-1α by the ubiquitin-proteasome system determines its prevalence [41]. In support of this notion, we demonstrated a preferential increase of association between SUMO-1 and HIF-1α in RVLM during the pro-life phase, and that immunoneutralization of SUMO-1 or Ubc9 significantly antagonized the augmented expression of HIF-1α in RVLM and exacerbated the dysfunction of brain stem cardiovascular regulation. We noted that the level of augmented HIF-1α detected from proteins immunoprecipitated by SUMO-1 during the pro-life phase (Figure 6A) was not overtly more than the increase in HIF-1α determined from total protein (Figure 3A). It is likely that this seeming discrepancy is a demonstration of the phenomenon of SUMO enigma [31], which stipulates that only a small proportion of the available substrate protein needs to be sumoylated to achieve maximal effects.

A well-known hypoxia responsive gene product is NOS II [19], [26]. Melillo et al. [50] showed that a sequence homologous to a hypoxia-responsive enhancer (NOS II-HRE) is responsible for activation of *nos II* gene in murine macrophages. A putative HIF-1 site (CTACGTGCT) in the murine NOS II gene was subsequently shown to be crucial for hypoxia-induced transcription in pulmonary artery endothelial cells [51] and cardiomyocytes [52]. Results from our previous studies [11]–[14] indicated that NOS II/peroxynitrite signaling in RVLM underlies the significant dysfunction of cardiovascular regulation seen during the pro-death phase of brain death. Since immunoneutralization of HIF-1α or HIF-1β did not significantly affect the progressive augmentation of NOS II or nitrotyrosine levels in ventrolateral medulla in our animal model, the participation of HIF-1 as a cellular signal upstream to NOS II/peroxynitrite pathways is deemed minimal. Our results did lend credence to the notion that HIF-1 acts as the upstream cellular signal to NOS I/PKG pathways. Hypoxia increases NOS I expression that parallels activation of HIF-1α in piglet ventricular tissues [17]. An increase in NOS I and HIF-1α expression also occurs in cerebral cortex of anemic rats [18]. The present study further showed that immunoneutralization of HIF-1α or HIF-1β blunted the surge of NOS I or PKG expression during Phase I Mev intoxication.

In conclusion, the present study provided novel demonstration that enhanced sumoylation and stabilization of HIF-1α in response to hypoxia in RVLM ameliorates the dysfunction of brain stem cardiovascular regulation in our Mev intoxication model of brain death via augmented expression selectively of the pro-life NOS I/PKG signaling cascade. It is generally contended that hypoxia serves as the culprit for many abnormal brain functions. Nonetheless, working in conjunction with a neural substrate whose neuronal activity is reflected in the waxing and waning of the life-and-death signal, it is intriguing that our results demonstrated that enhanced protein sumoylation in response to hypoxia in RVLM in fact ameliorates brain stem cardiovascular regulatory failure associated with experimental brain death. This information, along with the signaling cascades identified in the present study, should provide further mechanistic insights into the etiology of brain death and offer new directions for the development of therapeutic strategy against this fatal eventuality aiming at preventing brain stem cardiovascular regulatory failure.

## Materials and Methods

### Ethics Statement

All experimental procedures carried out in this study have been approved by the Laboratory Animal Committee of the Kaohsiung Chang Gung Memorial Hospital (CGMH97006), and were in compliance with the guidelines for animal care set forth by this Committee.

### Animals

Adult male Sprague-Dawley rats (264 to 345 g, n = 285) purchased from the Experimental Animal Center of the National Science Council, Taiwan were used. Rats received preparatory surgery under an induction dose of pentobarbital sodium (50 mg kg^−1^, i.p.). During the experiment, animals received continuous intravenous infusion of propofol (20–25 mg kg^−1^ h^−1^; Zeneca, Macclesfield, UK), which provided satisfactory anesthetic maintenance while preserving the capacity of central cardiovascular regulation [53]. They were allowed to breathe spontaneously with room air, and body temperature was maintained at 37°C by a heating pad.

### Mev intoxication model of brain death

SAP signals recorded from the femoral artery were subject to simultaneous on-line and real-time power spectral analysis [11]–[14], [25], [27]. We were particularly interested in the LF (0.25–0.8 Hz) component of the SAP spectrum because it takes origin from RVLM [8] and the waxing and waning of the LF power reflects the prevalence of the pro-life and pro-death phases during the progression towards brain death in patients who succumbed to organophosphate poisoning [10]. HR was derived instantaneously from SAP signals. Since Mev induces comparable cardiovascular responses when given systemically or directly to RVLM [16], we routinely microinjected Mev bilaterally into RVLM to elicit site-specific effects [11]–[14], [25], [27]. The only exception was when Mev was given intravenously in conjunction with evaluation of tissue hypoxia in RVLM. Temporal changes in pulsatile SAP, mean SAP (MSAP), HR and power density of the LF component were routinely followed for 180 min after the administration of Mev, in an on-line and real-time manner.

### Microinjection of test agents

Microinjection bilaterally of test agents into RVLM, each at a volume of 50 nl, was carried out stereotaxically and sequentially [11]–[14], [25], [27] via a glass micropipette connected to a 0.5-µl Hamilton microsyringe (Reno, NV). The coordinates used were: 4.5–5 mm posterior to lambda, 1.8–2.1 mm lateral to midline, and 8.1–8.4 mm below the dorsal surface of cerebellum. Test agents employed included Mev (kindly provided by Huikwang Corporation, Tainan, Taiwan) and aCSF that served as the vehicle control. A rabbit polyclonal antiserum against HIF-1α (Novus Biologicals, Littleton, CO), HIF-1β (Lifespan Biosciences, Seattle, WA), HIF-2α (Novus) or SUMO-1 (Cell Signaling, Danvers, MA); or a mouse polyclonal antiserum against Ubc9 (BD, San Jose, CA) was used to effect immunoneutralization. As in previous studies [11], [12], 0.02% Triton X-100 (Sigma-Aldrich, St. Louis, MO) was added to facilitate transport of the antiserum across the cell membrane of RVLM neurons. Microinjection of normal rabbit serum (NRS; Sigma-Aldrich) or normal mouse serum (NMS; Sigma-Aldrich) plus 0.02% Triton X-100 served as the vehicle control. To avoid verbose presentation, however, the phrase “0.02% Triton X-100” is omitted from subsequent narration. To avoid the confounding effects of drug interactions, each animal received only one antiserum pretreatment.

### Evaluation of tissue hypoxia in RVLM

A combined oxygen/temperature/blood flow probe (Oxford Optronix, Oxford, UK) stereotaxically positioned into RVLM was used in experiments that called for simultaneous and continuous measurement of tissue oxygen tension, temperature, and microvascular perfusion [15]. The dimension of the tip of the probe is approximately 800 µm. Instantaneous changes in local oxygen tension, compensated for fluctuations in tissue temperature, were processed by an OxyLite monitor (Oxford Optronix). Real-time microvascular red blood cell perfusion in tissue was processed by an OxyFlo monitor (Oxford Optronix). Laser Doppler signals from the tissue were recorded in blood perfusion units (BPU), which is a relative unit defined against a controlled motility standard.

### Collection of tissue samples

We routinely collected tissue samples [11]–[14], [25], [27] during the peak of the pro-life and pro-death phase (Mev group) or 30 or 180 min after microinjection of aCSF into RVLM (vehicle group). The brain was rapidly removed and placed on dry ice, and tissues from both sides of the ventrolateral medulla, at the level of RVLM (0.5 to 1.5 mm rostral to the obex) were collected by micropunches made with a stainless steel bore (1 mm i.d.) and frozen in liquid nitrogen. Medullary tissues collected from anesthetized animals but without treatment served as the sham controls. In some experiments, proteins from the nuclear or cytosolic fraction of the samples were extracted using a commercial kit (Active Motif, Carlsbad, CA). Total protein or that in the nuclear or cytosolic extracts was estimated by BCA Protein Assay (Pierce, Rockford, IL).

### Protein expression

We employed Western blot analysis [11]–[14], [25], [27] to detect changes in expression of HIF-1α, HIF-1β, HIF-2α, Ubc9, NOS I, PKG, NOS II or nitrotyrosine (marker for peroxynitrite) protein, or proteins conjugated by SUMO-1. The primary antiserum used for HIF-1α, HIF-1β, HIF-2α, SUMO-1 or Ubc9 were the same as those used for immunoneutralization. The other primary antisera used included a rabbit polyclonal antiserum against NOS I (Santa Cruz, Santa Cruz, CA), NOS II (Santa Cruz), PKG (Calbiochem, San Diego, CA); or a mouse monoclonal antiserum against nitrotyrosine (Upstate Biotechnology, Lake Placid, NY) or β-actin (Chemicon, Temecula, CA). The secondary antisera used included horseradish peroxidase-conjugated donkey anti-rabbit IgG (Gehealthcare, Uppsala, Sweden) for HIF-1α, HIF-1β, HIF-2α, SUMO-1, NOS I, NOS II, PKG; or horseradish peroxidase-conjugated sheep anti-mouse IgG (Gehealthcare) for Ubc9, nitrotyrosine or β-actin. The amount of protein was quantified by the ImageMaster software (Amersham Pharmacia Biotech, Buckinghamshire, UK), and was expressed as the ratio relative to β-actin protein. Densitometric values that were not statistically different from the background were designated below detection limits.

### Transcriptional activity of HIF-1

We measured HIF-1 DNA binding capacity [12] in nuclear proteins extracted from ventrolateral medulla using EMSA [54]. The 3′ end of a double-stranded synthetic oligonucleotide probe for HIF-1 (5′-ACCGGCCCTACGTGCT-GTCTCAC-3′ and 3′-TGGCCGGGATGCACGACAGAGTG-5′) [54] was labeled with digoxigenin-11-ddUTP (Roche, Penzberg, Germany). DNA and protein complexes resolved on 4% polyacrylamide gels by electrophoresis were detected by chemiluminescence after reacting with an anti-digoxigenin antiserum. Competitive assay using unlabeled HIF oligonucleotide served as the negative control. A rabbit polyclonal antiserum against HIF-1α or HIF-1β (Santa Cruz) was added to the DNA binding reaction cocktail in supershift assay to determine the participation of HIF subunits. The occurrence of nucleus-bound translocation of HIF-1α was confirmed using Western blot analysis on cytosolic or nuclear proteins [11], [12]. In some experiments, we quantified the transcription activity of HIF-1α using an ELISA-based HIF-1 transcription factor assay kit (Active Motif, Carlsbad, CA) [27]. In brief, HIF present in nuclear extract was allowed to bind to an oligonucleotide containing the hypoxia response element sequence (5′-TACGTGCT-3′). After the introduction of an antibody that recognizes an epitope on HIF-1α that is accessible on DNA binding, the addition of a secondary horseradish peroxidase-conjugated antibody provided a sensitive colorimetric readout that can be determined by spectrophotometry. HIF-1α activity was quantified by measuring the absorbance at 450 nm using an ELISA microtiter plate reader (Anthros Labtec, Salzburg, Austria), and was expressed as fold changes against sham controls.

### Immunofluorescence staining and confocal microscopy

We employed double immunofluorescence staining coupled with laser scanning confocal microscopy [11], [12] to detect nucleus-bound translocation or subcellular localization of HIF-1α, HIF-1β or SUMO-1 in RVLM neurons labeled with a mouse monoclonal antiserum against a specific neuron marker, neuron-specific nuclear protein (NeuN; Chemicon). Secondary antisera (Molecular Probes, Eugene, OR) used included a goat anti-rabbit IgG conjugated with Alexa Fluor 568 for HIF-1α or HIF-1β, and a goat anti-mouse IgG conjugated with Alexa Fluor 488 for NeuN; or a goat anti-rabbit IgG conjugated with Alexa Fluor 488 for SUMO-1 and a goat anti-mouse IgG conjugated with Alexa Fluor 568 for NeuN. Tissues similarly processed but omitting primary antiserum against HIF-1α, HIF-1β or SUMO-1 served as our negative controls. Immunoreactivity was viewed under a Fluorview FV10i laser scanning confocal microscope (Olympus, Tokyo, Japan).

### Immunoprecipitation and immunoblot analysis

We employed immunoprecipitation followed by immunoblot analysis [11], [12] to establish an interaction between HIF-1β and HIF-1α in nuclear proteins, or between SUMO-1 and HIF-1α in both cytosolic and nuclear proteins extracted from ventrolateral medulla. Protein extracts were immunoprecipitated with affinity-purified goat polyclonal anti-HIF-1β, rabbit polyclonal anti-SUMO-1 or mouse monoclonal anti-HIF-1α antiserum conjugated with protein G-agarose beads (Santa Cruz). Western blot analysis of HIF-1α or HIF-2α from proteins immunoprecipitated by anti-HIF-1β antiserum, HIF-1α from proteins immunoprecipitated by anti-SUMO-1 antiserum, or SUMO-1 from proteins immunoprecipitated by anti-HIF-1α antiserum was carried out as described above.

### Histology

In some animals that were not used for biochemical analysis, the brain stem was removed at the end of the physiological experiment and fixed in 10% formaldehyde-saline solution that contains 30% sucrose for at least 72 h. Frozen 25-µm sections of the medulla oblongata stained with neural red were used for histological verification of the microinjection sites.

### Statistical analysis

All values are expressed as mean ± SEM. The effects of various treatments on the averaged value of MSAP or HR calculated every 20 min after administration of test agents or vehicle, the sum total of power density for LF component in the SAP spectra over 20 min, or the protein expression level in the ventrolateral medulla during each phase of Mev intoxication, were used for statistical analysis. One-way or 2-way ANOVA with repeated measures was used, as appropriate, to assess group means. This was followed by the Scheffé multiple-range test for post hoc assessment of individual means. *P*<0.05 was considered to be statistically significant.
